# Constitutive Modeling of the Tensile Behavior of Recycled Polypropylene-Based Composites

**DOI:** 10.3390/ma12152419

**Published:** 2019-07-29

**Authors:** Kui Wang, Yong Peng, Rodrigue Matadi Boumbimba, Nadia Bahlouli, Daniel Pessey, Said Ahzi, Frédéric Addiego, Yves Rémond

**Affiliations:** 1 Key Laboratory of Traffic Safety on Track (Central South University) Ministry of Education, School of Traffic & Transportation Engineering, Central South University, Changsha 410075, China; 2 Joint International Research Laboratory of Key Technology for Rail Traffic Safety, Central South University, Changsha 410075, China; 3 Arts et Métiers ParisTech, LEM3, Université de Lorraine, CNRS, F-57000 Metz, France; 4 ICUBE Laboratory—CNRS, University of Strasbourg, 67000 Strasbourg, France; 5 Qatar Environment and Energy Research Institute (QEERI), Hamad bin Khalifa University (HBKU), Qatar Foundation, 34110 Doha, Qatar; 6 Department Materials Research and Technology (MRT), Luxembourg Institute of Science and Technology (LIST), ZAE Robert Steichen, 5 Rue Bommel, L‐4940 Hautcharage, Luxembourg

**Keywords:** polypropylene, composite, constitutive model, reprocessing, mechanical properties, strain rate

## Abstract

The effect of reprocessing on the quasi-static uniaxial tensile behavior of two commercial polypropylene (PP)-based composites is experimentally investigated and modeled. In particular, the studied materials consist of an unfilled high-impact PP and a talc-filled high-impact PP. These PP composites are subjected to repeated processing cycles, including a grinding step and an extrusion step to simulate recycling at the laboratory level, the selected reprocessing numbers for this study being 0, 3, 6, 9, and 12. Because the repeated reprocessing leads to thermo-mechanical degradation by chain scission mechanisms, the tensile behavior of the two materials exhibits a continuous decrease of elastic modulus and failure strain with the increasing amount of reprocessing. A physically consistent three-dimensional constitutive model is used to predict the tensile response of non-recycled materials with strain rate dependence. For the recycled materials, the reprocessing effect is accounted by incorporating the reprocessing sensitive coefficient into the constitutive model for Young’s modulus, failure strain, softening, and hardening equations. Our predictions of true stress—true strain curves for non-recycled and recycled 108MF97 and 7510—are in good agreement with experimental data and can be useful for industries and companies which are looking for a model able to predict the recycling effect on mechanical behavior of polymer-based materials.

## 1. Introduction

Polypropylene (PP)-based composites are increasingly utilized in the automotive industry, in particular, for both exterior and interior components. These materials have attractive mechanical properties, low density, excellent chemical resistance, and effectiveness price [1]. For manufacturing car bumpers tolerant to a high loading rate, elastomers, such as ethylene-propylene-copolymer (EPC) and ethylene-propylene-diene-monomer (EPDM), are generally mixed with neat PP to improve the impact properties [2]. However, these rubber particles lead to a decrease of stiffness and strength of the materials. In order to alleviate these decreases induced by the addition of rubber particles, mineral rigid reinforcements, such as talc, are usually added to the binary composite of the PP/elastomer [3].

The increasing use of PP-based composites results in huge plastic wastes from end-of-life vehicles (ELV), while the re-use and recovery of these wastes are quite limited [4]. For both environmental protection and waste management considerations, the European directive 2000/53/UE has been legislated to increase the re-use and recovery rate of these plastic wastes. Among the different end-of-life scenarios of plastic, the mechanical recycling is the simplest and most ecological one, where the post-used ELV plastics are mechanically crushed and re-processed to produce new structural parts [5].

It is well known that reprocessing at a high temperature, with intensive shearing conditions and with the presence of oxygen and impurities, could lead to thermal, thermo-oxidative, and thermo-mechanical degradations of the materials, affecting their properties. The mechanical reprocessing effects on the properties of PP-based composites have been studied [6,7]. Most of the previous studies focus on the characterization of rheological, chemical, physical, and mechanical properties of the materials with the number of reprocessing. Briefly, recycling induces a reduction of molecular weight that is attributed to chain scission mechanisms [8,9]. The lower molecular weight of recycled materials results in an increase in the crystallinity of the PP matrix [10,11]. The oxidative state of recycled composites depends on the presence of oxygen during the melted state and on the presence of anti-oxidative agents in the PP matrix [12]. Even though the reprocessed materials underwent critical reprocessing conditions, the decomposition temperature of the composite is little influenced by the reprocessing [13]. However, the evolution of the composite mechanical behavior during the mechanical recycling is not consistent in the open literature. The reasons for this are that the experimental conditions are drastically varying from one study to another one. On the other hand, the reprocessing not only leads to the degradation of the matrix but also changes the composite morphology and the filler properties. The reinforcement effects on the recycled PP-based composites have been recently investigated in our previous works [14,15,16,17]. We found that the ethylene octene copolymer (EOC) inclusions stabilize the tensile elongation at the break up to three recycling numbers due to a decrease of their size and the homogenization of their shape during the reprocessing. Otherwise, the talc fillers increase the stiffness and the strength of the composite with the number of recycling numbers due to a decrease of their geometry and an increase of their aspect ratio. Although the experimental characterization indicates the evolution of different properties of the composite during the mechanical recycling, the modeling of mechanical responses of recycled PP-based composites is less investigated. To this end, a micromechanical modeling may enable us to predict the maximum reprocessing number, until which the quality of the material would be retained for a targeted application.

In this work, the main objective is to experimentally determine and model the tensile behavior of two commercial PP-based composites as a function of the number of mechanical reprocessing. These two materials are commonly used for the manufacturing of car bumpers in the automotive industry, requiring optimizing their end-of-life scenarios and utilization. To simulate mechanical recycling, the materials were subjected to multiple reprocessing procedures, including a grinding step, an extrusion step, and an injection step. The experimental evaluation involves low strain rate uniaxial tensile testing up to failure. The analysis of the recycling-dependent tensile behavior has guided the development of a constitutive model, including the recycling correlative physical parameters.

## 2. Experimental Study

### 2.1. Materials and Processing

Two commercial grades of PP-based composites used for producing bumpers are studied in the present paper. The first grade was a two-phase material, referenced as a Sabic PP compound, grade 108MF97. It contained 22 wt.% of EPC inclusions whose diameter was lower than 1 μm. The second grade was a three-phase composite, referenced as a Sabic PP compound, grade 7510, which contained 12 wt.% of talc powder and 20 wt.% of EPDM particles. In this grade, the size of the talc fillers was around 8 μm and the diameter of the EPDM particles was less than 1 μm.

To simulate the procedure of mechanical recycling, the raw materials, initially in the form of pellets, were extruded by a twin-screw extruder (Clextral BC 21, Firminy, France) with a flow rate of 10 kg/h and at a final die temperature of 230 °C. The obtained extruded strands were quenched in a cold-water bath and were subsequently pelletized using a rapid granulator. The obtained pellets were dried in an air circulating oven to eliminate moisture for the next step. A total of 12 cycles of extrusion and pelletization was conducted by using the same machines under the same processing conditions. The following numbers of reprocessing were chosen: 0, 3, 6, 9, and 12. We note that 12 cycles of reprocessing can clearly show the recycling effect [5]. Finally, the pellets of selected materials were molded by an injection machine (Billion Visumat 1000, Bellignat, France) at 230 °C into plates of dimensions at 300 mm × 100 mm × 3 mm. Tensile samples (ASTM D638 type V) were carefully machined from the injected plates, along with the flow direction. In the following, these two PP-based composites were denoted with the number of extrusions as 108MF97 0P, 3P, 6P, etc. and 7510 0P, 3P, 6P, etc. It is to be noted that 0P is linked with the non-recycled materials.

### 2.2. Experimental Procedures

Tensile testing was performed on a universal material testing machine (MTS 810, MN, USA) with the Videotraction system at room temperature [18]. This system was developed to assess the true stress—the true strain tensile curve—based on the optical extensometer with a control of strain rate. Accordingly, all the materials were tested at a constant true strain rate of 0.001 s^−1^ until failure. In order to investigate the strain rate sensitivity of both materials, 108MF97 0P and 6P, 7510 0P and 6P were tested with additional strain rates of 0.01 s^−1^ and 0.0001 s^−1^. 

### 2.3. Experimental Results 

**Figure 1:**
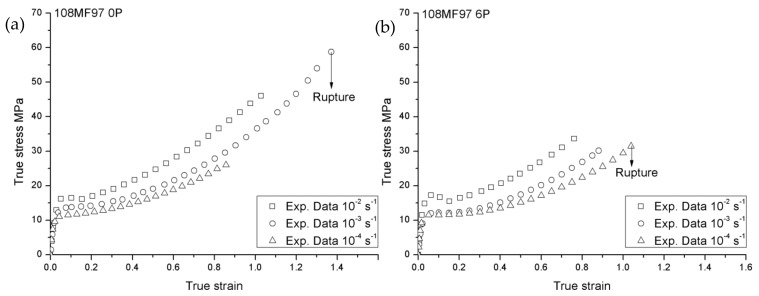
True stress vs. true strain responses of 108MF97 (**a**) 0P and (**b**) 6P, at three different strain rates.

**Figure 2:**
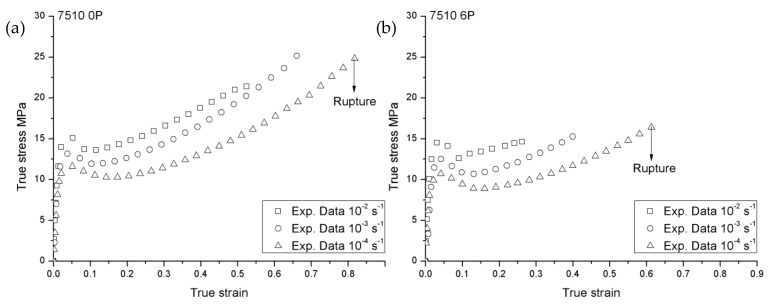
True stress vs. true strain responses of 7510 (**a**)0P and (**b**) 6P at three different strain rates.

Figure 1 and Figure 2 show true stress—true strain responses in the case of 108MF97 and 7510—at three different strain rates with the recycling number 0P and 6P, respectively. In these two figures, all the curves exhibit typical features of stress–strain curves, in which the curves can be divided into four parts: An initial linear elastic response, a visco-elastic transition to yield, a strain softening after the yielding point, and a strain hardening till to the failure [19]. In addition, in Figure 1 and Figure 2, all materials present a strain rate sensitivity in which the yield and flow stresses increase with the strain rates. The increase of strain rate leads to the hardening of the material due to less time for the molecular network to withstand the imposed strain. However, the mechanical responses at the highest strain rate show a greater post-yield strain softening, attributing mainly to the self-heating effect [20]. Indeed, during the plastic deformation, due to the low chain mobility coupled with internal friction, a large part of the plastic deformation work is converted into heat in the materials [21]. Comparing the mechanical responses of 108MF97 and 7510 (0P or 6P) at the same strain rates, the curves of 7510 show the more important post-yield strain softening. This is probably due to the presence of the talc filler. Indeed, the localized shearing deformation in the matrix, as well as at the filler/matrix interfaces, may generate much more heat during the plastic deformation [19]. 

Concerning the failure behavior of 108MF97 and 7510 in Figure 1 and Figure 2, the higher strain rate decreases the failure strain in the case of the two materials (0P or 6P, excepted for 108MF97 0P at the strain rate of 0.0001 s^−1^, due to the experimental error). The higher strain rate leads to fast crack propagation in the tensile sample and, consequently, a smaller failure strain of the materials [22]. Comparing 108MF97 and 7510 (Figure 1 and Figure 2), under the same strain rate and with the same recycling number, 108MF97 shows the larger ductility. Although the presence of elastomer particles has a toughening effect on both materials, the talc filler in 7510 may induce cavitation mechanisms by matrix/filler debonding. 

**Figure 3:**
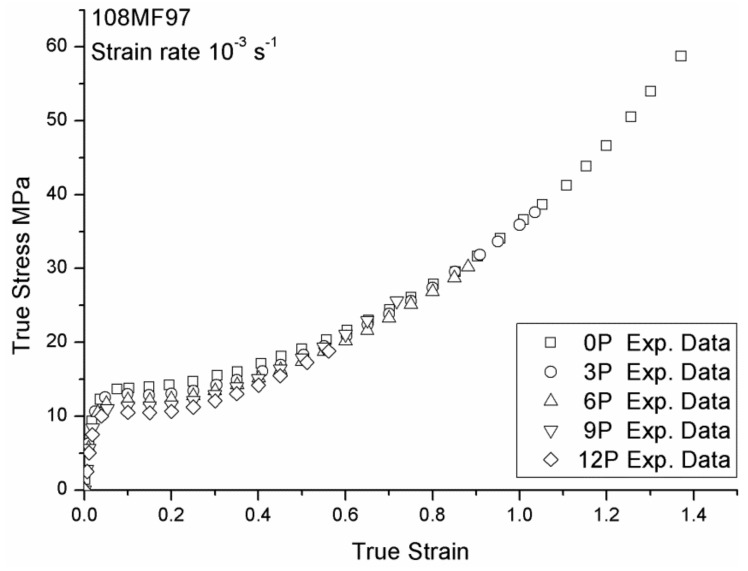
True stress vs. true strain responses of 108MF97 at different recycling numbers under a strain rate of 0.001 s^−^^1^.

**Figure 4:**
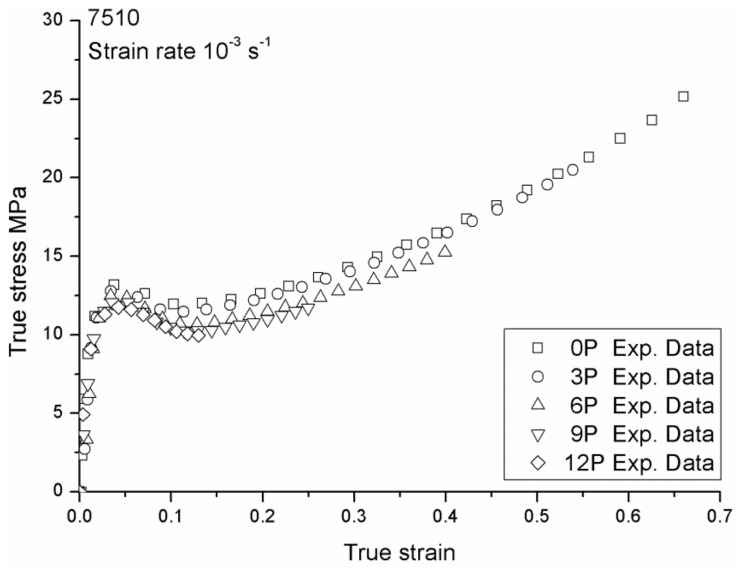
True stress vs. true strain responses of 7510 with different recycling numbers under a strain rate of 0.001 s^−1^.

The mechanical responses of 108MF97 and 7510 with different recycling numbers under strain rate 0.001 s^−1^ are shown in Figure 3 and Figure 4, respectively. These two figures show that the yield stress and Young’s modulus of both materials continuously decrease with the recycling numbers. Indeed, mechanical recycling conducted under intensive shearing at a high temperature, and with the presence of oxygen and impurities, generally leads to thermal, thermo-oxidative, and thermo-mechanical degradations of the materials. At the molecular level, the decrease of mechanical properties is mainly due to the chain scission mechanisms caused by the reprocessing [5]. Our previous studies found a decrease of average molecular weight with the recycling numbers for 108MF97 and a significant increase of the melt flow index (MFI) with a reprocessing number in the case of 7510. This increase of MFI also relates to a decrease of molecular weight of the PP matrix for 7510 [5]. In fact, the mechanical recycling shortens polymer chains and, hence, increases chain mobility. This higher mobility can result in an increase of crystallinity content of the PP matrix and, consequently, can stiffen materials [15]. As shown in Figure 3 and Figure 4, the stiffer recycled materials show a greater strain hardening effect than the non-recycled materials. This effect is more significant for 108MF97. In addition, the failure strain has a continuous decrease with the reprocessing number for both materials. This is due to the shortening of the molecular chains of the PP matrix, inducing disentanglement in the amorphous phase and decreasing the inter-connection between the crystalline phase and the amorphous phase of materials. As a result, the failure strain of both materials decreases with the reprocessing number.

## 3. Constitutive Relation

The constitutive model considered in this work was based on the previous work of Wu and Ahzi [23]. In their work, the authors developed a physically consistent model to describe the strain rate and temperature dependency of the large inelastic deformation response of non-filled and silica-filled cured resin. They used a molecular theory for the plastic flow and an orientational model for the deformation of polymer chains at a large strain rate. In our study, we have chosen to describe the elastic behavior of materials by the strain rate dependent equation of Zhou et al. [24]. The yield and post-yield behavior was described by the strain rate and temperature dependent flow rule of Boyce et al. [25]. The orientational hardening was modeled through the eight-chains rubber-elasticity law [26].

### 3.1. Strain Rate Dependent of Young’s Modulus and Failure Strain

Non-recycled 108MF97 and 7510 are strain-rate-sensitive materials (Figure 1 and Figure 2). The linear relationships between Young’s modulus (E) and the natural logarithm strain rate $\ln\left(\dot{\varepsilon }\right)$, and between the failure strain $\varepsilon_{R}$ and $\ln\left(\dot{\varepsilon }\right),$ can be described as Equations (1–2) [24]:

(1) $$E\left(\dot{\varepsilon }\right)=E_{0}\left(1+\lambda_{E}\cdot \ln\frac{\dot{\varepsilon }}{\dot{\varepsilon_{0}}}\right)$$

(2) $$\varepsilon_{R}\left(\dot{\varepsilon }\right)=\varepsilon_{R_{0}}\left(1+\lambda_{\varepsilon_{R}}\cdot \ln\frac{\dot{\varepsilon }}{\dot{\varepsilon_{0}}}\right)$$

where $E_{0}$, $\varepsilon_{R_{0}}$, ${\dot{\varepsilon }}_{0}$ are the reference elastic modulus, reference failure strain, and reference strain rate of non-recycled materials, respectively. The parameters $\lambda_{E}$ and $\lambda_{\varepsilon_{R}}$ are defined as the strain rate strengthening coefficient of Young’s modulus and the failure strain of non-recycled materials, respectively.

### 3.2. Flow Rule

For large elastic-viscoplastic deformation of glassy polymers, Boyce et al. [25] extended Argon’s model and proposed a three-dimensional constitutive model. This model contains the yield and post-yield behavior of the material with the pressure, strain rate, and temperature dependency. The extended model is expressed as Equation (3) [25]:

(3) $${\dot{\gamma }}^{P}={\dot{\gamma }}_{0}exp\left[-\frac{AS_{0}}{T}\left(1-{\left(\frac{\tau }{S_{0}}\right)}^{\frac{5}{6}}\right)\right]\mathrm{with}\;A=\frac{39\pi \omega^{2}\alpha^{3}}{16k}$$

where ${\dot{\gamma }}^{P}$ is the plastic strain rate, ${\dot{\gamma }}_{0}$ is the pre-exponential shear strain rate factor, ω is the net angle of rotation of the molecular segment, α is the mean molecular radius, and k is the Boltzmann constant. T is the absolute temperature and τ is the shear yield strength. The athermal shear yield strength of the material ($S_{0}$) at absolute 0 is approximately given by Equation (4):

(4) $$S_{0}=\frac{0.077\mu }{1-\upsilon }$$

Here, μ is the shear modulus and υ is the Poisson’s ratio of the material.

### 3.3. Strain Softening

In the constitutive model of Boyce et al. [25], strain softening is incorporated into the constitutive law by modifying the athermal yield strength, $S_{0}$ of the material as plastic straining occurs. It is based on the assumption that as the material begins to undergo plastic deformation, there is some average restructuring of the molecular chains in the flow state that causes an actual fall in the athermal shear resistance of the material. Thus, the phenomenological evaluation of the shear resistance during strain softening is defined as Equation (5):

(5) $$\dot{S}=h\left(1-\frac{S}{S_{ss}}\right){\dot{\gamma }}^{P}$$

where $\dot{S}$ is the softening evolution of the shear resistance, h is the softening slope, S is is the current athermal deformation resistance of the material, and $S_{ss}$ is the resistance referring to the preferred structure determined by the temperature and strain rate [27].

### 3.4. Orientational Hardening

The hardening effect is determined by computing the external stress state that results when the material is stretched at temperatures above its glass transition (*T_g_*) and assuming that this state represents an internal resistance which is locked in the material. The locked internal resistance related to an applied external stress state is therefore named the back stress of materials when undergoing deformation. Arruda and Boyce developed an eight-chain model to describe this network stress tensor as Equation (6) [26]:

(6) $$B_{i}=\frac{C_{R}}{3}\sqrt{N}L^{-1}\left(\frac{\lambda_{chain}}{\sqrt{N}}\right)\frac{\lambda_{i}^{P^{2}}-\frac{1}{3}I_{1}}{\lambda_{chain}}$$

where $B_{i}$ is a principal value of the deviatoric back stress tensor, the subscript i = 1–3 designates one of the three principal directions. $C_{R}$ is a temperature-dependent rubber modulus [28], N is the number of rigid links of the entanglement of chains, $\lambda_{chain}={\left(\left(\lambda_{1}^{2}+\lambda_{2}^{2}+\lambda_{3}^{2}\right)/3\right)}^{1/2}$ and $\lambda_{i}$ are the imposed principal stretch, $\lambda_{i}^{P}$ is the principal values of the left Cauchy stretch tensor $V_{p}$, and the subscript *p* designates the plastic part. $I_{1}$ is the stretch tensor with $I_{1}=\lambda_{1}^{2}+\lambda_{2}^{2}+\lambda_{3}^{2}$. The Langevin function is defined by $L\left(y\right)=\coth\left(y\right)-\frac{1}{y}=x$, with its inverse form $L^{-1}\left(x\right)=y$.

### 3.5. Recycling Effect

As shown in Figure 3 and Figure 4, Young’s modulus, *E* and failure strain, $\varepsilon_{R}$ of 108MF97 and 7510 have a continuous decrease with the reprocessing number. Thus, we have assumed a linear evolution of these two parameters as shown in Equation (7–8) with strain rate and reprocessing dependencies, based on Equations (1) and (2):

(7) $$E\left(N_{P,}\dot{\varepsilon }\right)=\left(k_{1}N_{P}+E_{0}\right)\cdot \left(1+\left(\xi_{1}\cdot N_{P}+\lambda_{E}\right)\cdot ln\frac{\dot{\varepsilon }}{\dot{\varepsilon_{0}}}\right)$$

(8) $$\varepsilon_{R}\left(N_{P,}\dot{\varepsilon }\right)=\left(k_{2}N_{P}+\varepsilon_{R_{0}}\right)\cdot \left(1+\left(\xi_{2}\cdot N_{P}+\lambda_{\varepsilon_{R}}\right)\cdot ln\frac{\dot{\varepsilon }}{\dot{\varepsilon_{0}}}\right)$$

where $N_{P}$ is the number of reprocessing and $k_{1}$ and $k_{2}$ are the reprocessing strengthening coefficient of Young’s modulus and failure strain, respectively. $\xi_{1}$ and $\xi_{2}$ are the recycling strengthening coefficient of the strain rate. For the non-recycled materials, $N_{P}$ is equal to 0 and Equations (7) and (8) reduce to the same form of Equations (1) and (2).

The mechanical recycling induces microstructure changes of the materials, i.e., the chain length and the crystallinity content. With the need to exhibit mathematical simplicity, we have assumed a linear evolution of $C_{R}$, *N*, and $R_{s}$ with a reprocessing number as Equations (9–11):

(9) $$C_{R}\left(N_{p}\right)=k_{3}N_{P}+C_{R_{0}}^{ref}$$

(10) $$N\left(N_{p}\right)=k_{4}N_{P}+N_{0}^{ref}$$

(11) $$R_{s}\left(N_{p}\right)=k_{5}N_{P}+R_{S_{0}}^{ref}$$

where $R_{s}$ is the ratio of *S_ss_*/*S*_0_ and *k*_3_, *k*_4_, and *k*_5_ are the reprocessing strengthening coefficients of these three parameters. $C_{R_{0}}^{ref}$, $N_{0}^{ref}$, and $R_{S_{0}}^{ref}$ are the reference parameters for the non-recycled materials.

## 4. Constitutive Modeling

### 4.1. Kinematics of Finite Strain

The kinematics and kinetics of the finite deformation elastic-viscoplastic model for our materials are briefly presented below. The elastic-plastic deformation gradient tensor *F* is multiplicatively decomposed into two parts as Equation (12) [23]:

(12) $$F=F^{e}F^{P}$$

where the subscript *e* and *p* designate the elastic and plastic part, respectively.

The velocity gradient *L* and the plastic velocity gradient $L^{P}$ are given by Equation (13–14):

(13) $$L=\dot{F}F^{-1}={\dot{F}}^{e}F^{e^{-1}}+F^{e}L^{P}F^{e^{-1}}$$

and

(14) $$L^{P}={\dot{F}}^{P}F^{P^{-1}}≅D^{P}$$

where $D^{P}$ is the plastic strain rate gradient, while the plastic spin is considered equal to 0.

Polar decomposition of the plastic deformation gradient is shown in Equation (15):

(15) $$F^{P}=V^{P}R^{P}$$

and the Cauchy stress tensor is given by Equation (16):

(16) $$T=\frac{1}{J}C^{e}\left(lnF^{e}\right)$$

where *J* is volume change and $C^{e}$ is the elastic constant of the fourth order tensor.

The driving stress tensor is expressed as Equation (17):

(17) $$T^{*}=T-\frac{1}{J}F^{e}BF^{e^{T}}$$

where *B* is the back-stress tensor given previously.

The rate of plastic straining is constitutively described by Equation (18):

(18) $$D^{P}={\dot{\gamma }}^{P}N$$

where the plastic strain rate, ${\dot{\gamma }}^{P}$ is defined in Equation (2).

The resulting normalized deviatoric portion of the driving stress tensor is given by Equation (19):

(19) $$N=\frac{1}{\sqrt{2}\tau }T^{*’}$$

where the effective equivalent stress $\tau ={\left(T^{*’}\cdot T^{*’}/2\right)}^{1/2}$. Here, $T^{*’}$ is the deviatoric part of the driving stress tensor. Knowing the equivalent strain and the equivalent stress, the equivalent stress versus the equivalent strain curves are fitted and compared with the experimental data.

### 4.2. Modeling Results

In this constitutive model, several material constants need to be identified experimentally. First, the strain rate dependent on Young’s modulus and failure strain of the non-recycled 108MF97 and 7510 are measured using the experimental true stress—the true strain curves for non-recycled state under the strain rate 0.01 s^−1^ and 0.001 s^−1^ in Figure 1 and Figure 2. The reference elastic modulus (*E_0_*), the reference failure strain ($\varepsilon_{R_{0}}$), and the strain rate strengthening coefficient $\lambda_{E}$ and $\lambda_{\varepsilon_{R}}$ in Equations (1) and (2) can be determined by a reference strain rate of 1 s^−1^. 

Once the Young’s modulus is determined, the athermal shear yield strength of materials, *S_0_*, in Equation (4) can be computed with the Poisson ratio for the 108MF97 and 7510 as 0.31 and 0.3, respectively [5]. The values of the A (*K*/*MPa*) and ${\dot{\gamma }}_{0}$ (s^−1^) for non-recycled materials are obtained from the experimental yield stresses in Figure 1 and Figure 2, by using Equation (3).

Concerning the parameters of the softening effect, by comparing the maximum with the minimum yield stress under a strain rate of 0.001 s^−1^ during softening in Figure 1 and Figure 2 for two non-recycled materials, the ratios *S_ss_*/*S*_0_ were equal to 0.975 and 0.911 for 108MF97 and 7510, respectively. Thus, the steady state shear resistance can be calculated by using *S_ss_ = S*_0_ * 0.975 for non-recycled 108MF97 and *S_ss_* = *S*_0_ * 0.911 for non-recycled 7510. Consequently, the rate of the yield drop is measured by $h≅S_{ss}/\Delta \gamma^{P},$ where $\Delta \gamma^{P}$ is the strain between the maximum and the minimum yield stresses under a strain rate of 0.001 s^−1^.

**Figure 5:**
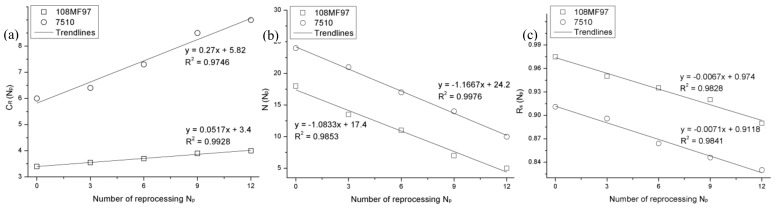
Reprocessing parameters (**a**) *C_R_*, (**b**) *N*, and (**c**) $R_{s}$ for 108MF97 and 7510.

Concerning the recycling effect parameters, first, Young’s moduli and failure strains are measured from the experimental curves in Figure 3 and Figure 4 for 108MF97 and 7510 with different numbers of reprocessing. Other parameters, such as *C_R_*, *N*, and *R_s_* are selected according to the experimental true stress—the true strain curves. The reprocessing sensitive parameters are reported in Table 1. Once these parameters are experimentally determinated, the reprocessing strengthening coefficients for Young’s modulus and failure strain in Equations (7) and (8) can be calculated. The reprocessing strengthening coefficient and the reference parameters of *C_R_*, *N*, and *R_s_*, in Equations (9)–(11) can be chosen by their trendlines, which yield the relations with the numbers of reprocessing as shown in Figure 5.

As reported by Arruda et al. [28], the rubber modulus, *C_R_*, is taken to be proportional to the chain density (*n*): *C_R_* = *nkT*, where *k* is the Boltzmann constant and *T* is the absolute temperature. In Figure 5, *C_R_* increases with the reprocessing number. This is due to the fact that reprocessing increases the crystallinity content of materials and, consequently, increases the polymer chain density [29]. This increase of crystallinity content of the matrix stiffens materials that generate more heat during the plastic deformation. This is probably the main reason for the decrease of the ratio, *R_s_*, with the reprocessing number for both composites. Contrarily, the number of rigid links in the entanglement of chains, *N*, decreases with the increasing of the reprocessing cycle. Because the repeated reprocessing gradually shortens the molecular chains, and decreases the chain entanglements [14]. All the coefficients and the reference parameters used for the modeling are summarized in Table 2. 

**Figure 6:**
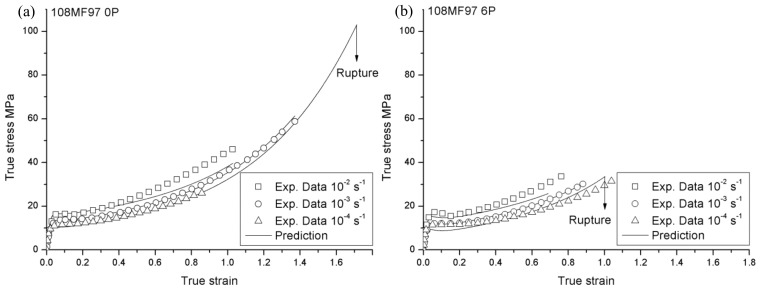
Comparison between the experimental and the predicted tensile behavior of 108MF97 (**a**) 0P and (**b**) 6P at three different strain rates.

**Figure 7:**
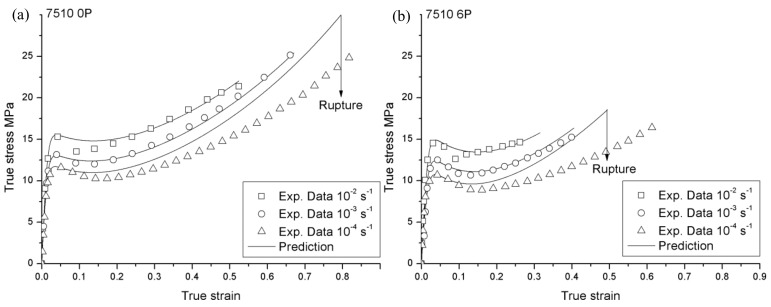
Comparison between the experimental and the predicted tensile behavior of 7510 (**a**) 0P and (**b**) 6P at three different strain rates.

The modeling results are compared to the experimental data for 108MF97 and 7510 at three different strain rates in Figure 6 and Figure 7. In our simulation, for the purpose of mathematical simplicity as mentioned previously, we considered that the physic meaning parameters, *C_R_*, *N*, and *R_s_*, are not depending on the strain rate and they are selected in accordance with the experimental curves at a strain rate of 0.001s^−1^. Thus, the predictions of the hardening part of the true stress—the true strain curves under other strain rates—have dispersions with the experimental data for both materials. In fact, different strain rates could generate different heat during the plastic deformation. The elevation of temperature affects the *R_s_* and the rubber modulus, *C_R_*, which is a temperature dependence parameter [28,30]. Besides that, our predictions have good correlation with experimental data.

**Figure 8:**
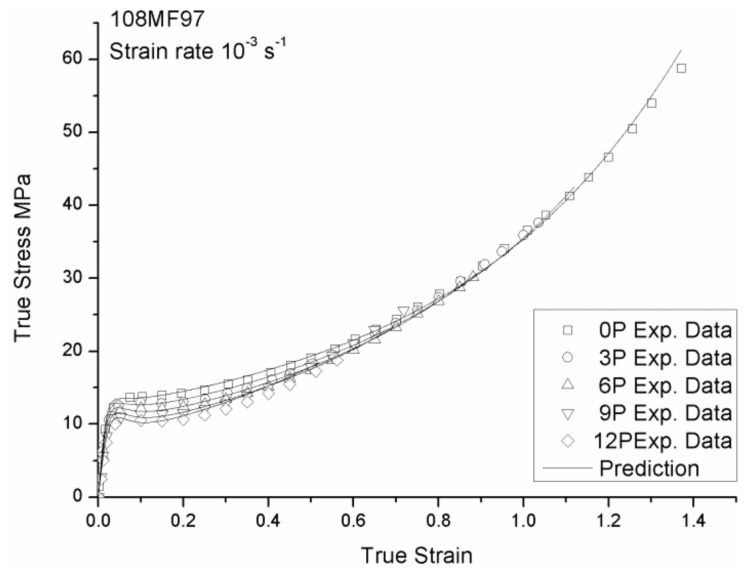
Comparison between the experimental and the predicted tensile behavior of the non-recycled and recycled 108MF97 under the strain rate of 0.001s^−1^.

**Figure 9:**
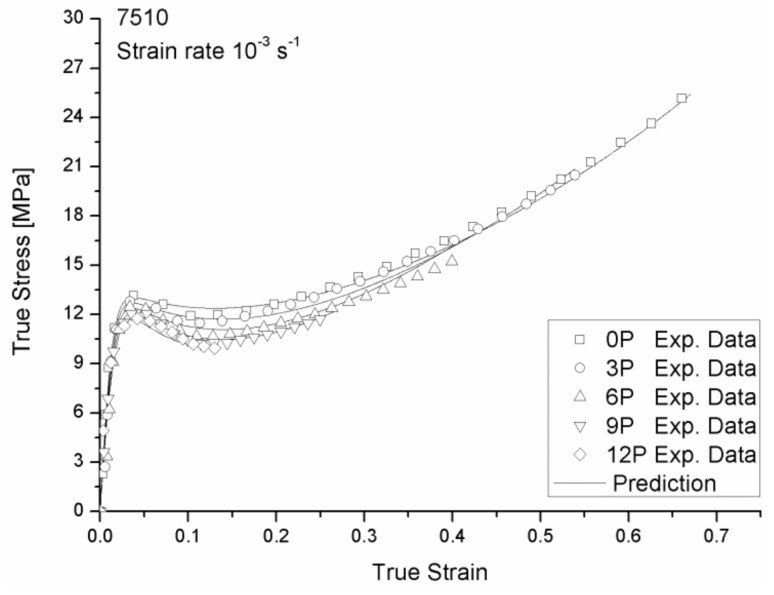
Comparison between the experimental and the predicted tensile behavior of the non-recycled and recycled 7510 under the strain rate of 0.001s^−1^.

Figure 8 and Figure 9 show good agreement between the experimental data and the modeling of the true stress—the true strain curves for the 108MF97 and 7510 with different reprocessing numbers under the strain rate of 0.001s^−1^, respectively. As shown in Figure 8 and Figure 9, the use of the strain rate and reprocessing dependent equations for Young’s modulus and failure strain provides a correct description of the tensile behavior of our materials at the small strain and at the largest strain, respectively. The incorporated strain softening and hardening effects can reasonably predict the experimental curves for non-recycled materials. The use of reprocessing-dependent equations for the rubber modulus, *C_R_*, the number of rigid links of the polymer chain, *N*, and the ratio of *S_ss_*/*S*_0_ and *R_s_*, can well predict the change of strain softening and hardening effects with the number of reprocessing for the recycled materials.

## 5. Conclusions

In order to investigate the effect of mechanical reprocessing on the mechanical properties of PP-based composites that are usually used for the manufacturing of car bumpers, two commercially available PP-based composites (PP/elastomer, PP/elastomer/talc) were re-grinded and re-extruded up to 12 times. Their mechanical characterizations were performed by quasi-static uniaxial tensile testing in the case of the selected recycling numbers 0, 3, 6, 9, and 12. By using the Videotraction technique, the true stress—true strain curveswere recorded till to the failure of materials. Due to the presence of the talc filler in 7510, this composite has higher Young’s moduli but lower failure strains, compared to 108MF97 with the same recycling number and under the same strain rate. The mechanical properties, such as yield stress, Young’s modulus, and failure strain of both materials, decrease with the increasing reprocessing number. This is because the mechanical reprocessing at a high temperature and intensive shearing conditions has led to thermo-mechanical degradations of the materials by the chains scission mechanism. For the interest of predicting the mechanical properties of the non-recycled and recycled materials, a three-dimensional constitutive model was used. In this model, the reprocessing strengthening coefficients were incorporated into the constitutive equation for Young’s modulus, failure strain, and the softening and hardening effects. Our model predictions are compared to the experimental results from the non-recycled and recycled composites, and a good agreement has been found. The proposed model can be seen as a major step towards the development of constitutive models for recycled polymer-based materials.

## Figures and Tables

**Table 1:** Reprocessing sensitive parameters.

N_p_	E (MPa)		ε_R_		C_R_		N		R_s_	
	108MF97	7510	108MF97	7510	108MF97	7510	108MF97	7510	108MF97	7510
0	850	1100	1.37	0.66	3.4	6	18	24	0.975	0.911
3	790	1050	1.04	0.54	3.55	6.4	13.5	21	0.95	0.896
6	730	1000	0.88	0.40	3.7	7.3	11	17	0.935	0.864
9	650	950	0.72	0.25	3.9	8.5	7	14	0.92	0.846
12	590	920	0.56	0.13	4	9	5	10	0.89	0.83

**Table 2:** The coefficients and the reference parameters for modeling.

	$k_{1}$	$k_{2}$	$k_{3}$	$k_{4}$	$k_{5}$	$E_{0}^{ref}$	$\varepsilon_{R0}^{ref}$	$C_{R0}^{ref}$	$N_{0}^{ref}$	$R_{s0}^{ref}$	$\xi_{1}$	$\xi_{2}$	$\lambda_{E}^{ref}$	$\lambda_{\varepsilon_{R}}^{ref}$
108MF97	–31.11	–0.01	0.05	–1.08	–0.01	977.51	0.34	3.4	17.4	0.97	–0.001	0.02	0.019	–0.43
7510	–9.87	–0.02	0.27	–1.17	–0.01	1250	0.23	5.82	24.2	0.91	0.001	–0.001	0.017	–0.28
